# Recent Advancements in Two-Dimensional Layered Molybdenum and Tungsten Carbide-Based Materials for Efficient Hydrogen Evolution Reactions

**DOI:** 10.3390/nano12213884

**Published:** 2022-11-03

**Authors:** K. Karuppasamy, A. Nichelson, Dhanasekaran Vikraman, Jun-Hyeok Choi, Sajjad Hussain, C. Ambika, Ranjith Bose, Akram Alfantazi, Hyun-Seok Kim

**Affiliations:** 1 Division of Electronics and Electrical Engineering, Dongguk University-Seoul, Seoul 04620, Korea; 2 Department of Physics, National Engineering College, K.R. Nagar, Kovilpatti, Tuticorin 628503, Tamil Nadu, India; 3 Department of Nanotechnology and Advanced Materials Engineering, Sejong University, Seoul 05006, Korea; 4 Department of Physics, Ayya Nadar Janaki Ammal College, Sivakasi 626123, Tamil Nadu, India; 5 Department of Chemical Engineering, Khalifa University, Abu Dhabi 127788, United Arab Emirates; 6 Emirates Nuclear Technology Center (ENTC), Khalifa University, Abu Dhabi 127788, United Arab Emirates

**Keywords:** two-dimensional nanostructures, tungsten carbide, energy conversion, hydrogen evolution reaction, Mo_x_C electrocatalysts

## Abstract

Green and renewable energy is the key to overcoming energy-related challenges such as fossil-fuel depletion and the worsening of environmental habituation. Among the different clean energy sources, hydrogen is considered the most impactful energy carrier and is touted as an alternate fuel for clean energy needs. Even though noble metal catalysts such as Pt, Pd, and Au exhibit excellent hydrogen evolution reaction (HER) activity in acid media, their earth abundance and capital costs are highly debatable. Hence, developing cost-effective, earth-abundant, and conductive electrocatalysts is crucial. In particular, various two-dimensional (2D) transition metal carbides and their compounds are gradually emerging as potential alternatives to noble metal-based catalysts. Owing to their improved hydrophilicity, good conductivity, and large surface areas, these 2D materials show superior stability and excellent catalytic performances during the HER process. This review article is a compilation of the different synthetic protocols, their impact, effects of doping on molybdenum and tungsten carbides and their derivatives, and their application in the HER process. The paper is more focused on the detailed strategies for improving the HER activity, highlights the limits of molybdenum and tungsten carbide-based electrocatalysts in electro-catalytic process, and elaborates on the future advancements expected in this field.

## 1. Introduction

Developing clean, sustainable, and affordable energy systems with abundant, non-toxic, and inexpensive materials for the future is a global challenge [1]. Hydrogen, a light element with a high energy density, is the cleanest alternative to fossil fuels [2,3] and is broadly investigated for application in green-energy technologies. The formation of hydrogen gas through electrolysis of water is termed hydrogen evolution reaction (HER), which has gained considerable attention in recent years owing to their various catalytic applications, especially in photoelectrochemical and electrochemical water-splitting [4,5]. For HER, robust, efficient, cost-effective, and earth-abundant electrocatalysts that demonstrate superior electrochemical activity are required. Platinum is a promising electrocatalyst for HER despite being expensive and scarce. In general, electrocatalysis is defined as a kind of catalysis that results in the alteration of the rate of electrochemical reactions that occurs on the electrode surface. The electrochemical performance of HERs strongly depend on the hydrogen adsorption configurations, chemical composition, and surface functionalization [6]. Researchers around the world are determined to identify a suitable non-platinum electrocatalyst that is non-precious, durable, earth-abundant, and stable. Earth-enriched materials, such as electrocatalysts, are not feasible for large-scale hydrogen production for industrial applications. Scientists have employed density functional theory calculations to identify superior electrocatalysts for HERs to produce hydrogen energy. An efficient electrocatalyst exhibits high electrocatalytic activity, low overvoltage, good electrochemical stability, high exchange current density, and decreased Tafel slope, and is cost effective for HERs as well [7]. The performance of HER electrode materials could be improved by optimizing the geometrical aspects (such as the specific surface area), forming core-shell nanostructures, nanoengineering electrodes, modifying the structure and chemical composition, and tuning the electronic structure of the electrode materials by doping cations and anions into the crystal lattice of the material. The intrinsic catalytic activity can be improved by doping and alloying the metal elements with non-metal elements. Over the past few decades, many efforts have been made to find highly active and long-lasting electrocatalyst materials with optimized characteristics for HERs in alkaline and acid media to realize an efficient hydrogen production [8,9,10,11]. To date, several precious and non-precious metal elements and their alloys, carbon-based materials, transition metal-based sulfides, phosphides, dichalcogenides, carbides, nitrides, and phosphosulfates have been investigated for HERs [1,12,13]. Recently, transition metal carbides (TMCs), transition metal nitrides (TMNs), and carbonitrides have been exploited for HERs because of the rich chemistry of these MXene compounds, including a high electrical conductivity, strong hydrophilicity, and surface functionalization. These catalysts have been proven highly efficient for HER. Among the known TMCs, molybdenum and tungsten carbides display substantial catalytic activities. The MXene-based compounds Mo_2_CT_x_ and Ti_2_CT_x_ have been investigated both computationally and experimentally as HER catalysts. The catalytic activity of Mo_2_CT_x_ is higher than that of Ti_2_CT_x_ [14,15,16]. Density functional theory calculations predicted that Mo_2_CT_x_ can be categorized into three separate classes based on their hydrogen adsorption properties, and in all these categories, the hexagonal close-packed (hcp) site is the lowest free energy adsorption site for oxygen for both the nitride and carbide (M = Mo, W, and Cr). For conventional HER electrolyzers, different non-noble metal catalysts have been widely studied in the past [17,18]. In majority of these studies, nickel- and cobalt-based compounds were used as the potential catalysts for HER. However, the stability of these compounds under neutral pH conditions is limited [19,20]. Tungsten carbide is a plausible earth-abundant catalyst that is cost effective and non-toxic, and shows a promising HER catalytic activity [17,19,21,22]. Further, this particular catalyst exhibits advantageous catalytic composition, activity, and stability under neutral pH conditions.

Nevertheless, the number of topical reviews on the catalytic activity of Mo_x_C and WC/W_2_C is limited, and thus, a detailed and systematic review of the current advancements in molybdenum- and tungsten-based electrocatalysts for HER activity is necessary at this juncture. This review is focused on the current progresses made in two-dimensional (2D) layered TMCs, especially molybdenum and tungsten carbides, for HER in acidic and alkaline solutions. The basic principles, mechanism, and various electrochemical parameters of HERs are elucidated here. State-of-the-art catalyst design and fabrication strategies for Mo_2_C and tungsten carbide (WC, W_2_C)-based catalysts for HER are presented. Finally, we discuss the next generation of Mo_2_C and tungsten carbide (WC, W_2_C)-based HER catalysts and their role in green hydrogen production and realizing a sustainable energy future.

## 2. Basic Principles of Electrocatalytic HER

**Figure 1:**
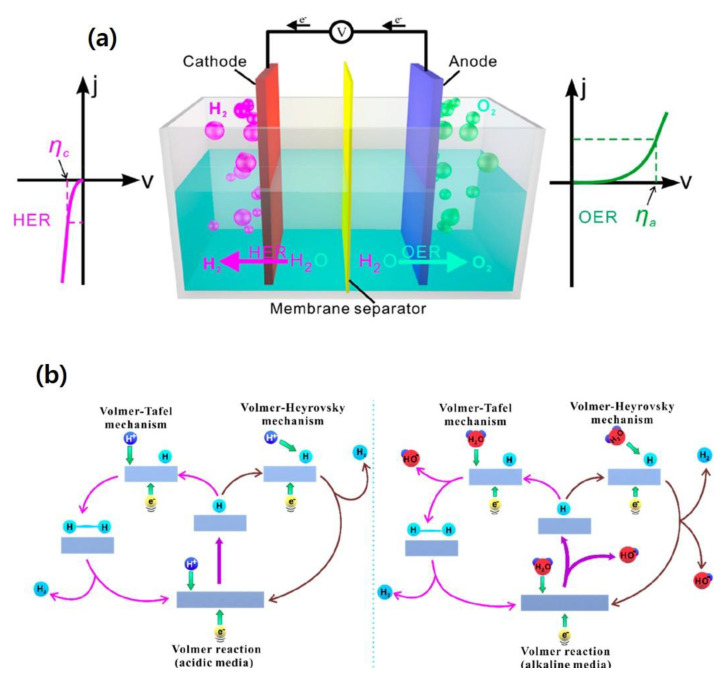
(**a**) Schematic mechanism of OER and HER processes during overall water-splitting process, (**b**) fundamental principles of HER over the surface of electrocatalysts in acidic and alkaline environments [23]. Copyright 2020 The American Chemical Society.

Electrocatalytic water-splitting (EWS) is a widely used process that transforms electrical energy into hydrogen fuel, with natural water as the prime source and oxygen and hydrogen as the byproducts. During the ESW process, two types of half-cell reactions occur, viz. evolution of oxygen at the anode, namely oxygen evolution reaction (OER) and production of hydrogen at the cathode, namely HER. The overall EWS process is demonstrated in Figure 1a. Several experimental and theoretical studies have been conducted on the HER mechanism, in alkaline and acidic media through the Volmer–Tafel or Volmer–Heyrovsky routes as displayed in Figure 1b [23]. When HER occurs in an acidic solution, first, hydrogen is electrochemically adsorbed over the electrode surface, followed by the generation of molecular hydrogen due to desorption [24]. In the final step, the adsorbed hydrogen is transformed into *H*_2_ as follows, Equations (1)–(3):

(1) $$e^{-}+H^{+}\to H^{*}\;(\mathrm{Volmer})\;$$

(2) $$e^{-}+H^{+}+H^{*}\to H_{2}\;(\mathrm{Heyrovsky})\;$$

(3) $$H^{*}+H^{*}\to H_{2}\;(\mathrm{Tafel})\;$$

When HER occurs in an alkaline solution, the water molecules dissociate to form protons, which involves the Volmer and Heyrovsky steps (Figure 1b). Next, unlike the acidic solution, no change occurs in the Tafel step as demonstrated in Equations (4)–(6) [25],

(4) $$H_{2}O+e^{-}\to OH^{-}+H^{*}\;(\mathrm{Volmer})\;$$

(5) $$e^{-}+H^{*}+H_{2}O\to H_{2}+OH^{-}\;(\mathrm{Heyrovsky})$$

(6) $$2e^{-}+2H_{2}O\to H_{2}+2OH^{-}\;(\mathrm{Tafel})$$

### 2.1. Parameters Governing the Electrocatalytic HER Process

In order to understand the electrocatalytic process of HERs, several significant parameters need to be determined, including the onset potential or overpotential (η), Gibb’s free energy, turnover frequency (TOF), faradaic efficiency, Tafel slope, exchange current density, and stability. These parameters provide insights into the performance of the catalysts.

#### 2.1.1. Overpotential η

During water electrolysis, the accumulation of H_2_ over the surface of the electrode hinders the electrode reactions, resulting in a sluggish reaction rate. In order to overcome this issue, an addition potential called the overpotential (η) could be applied during the electrolysis process. Overpotential is basically the difference between the thermodynamic potential for a given HER and the applied potential at which the catalyst operates at a specific current under specific conditions. In general, the water electrolysis equation has three major components (Equation (7)), i.e., the overpotential Δ*E_irreversible_*, the theoretical disintegration potential *E_reversible_*, and the dropped voltage IR [26].

(7) $$E_{electrolysis}\to E_{reversible}+\Delta E_{irreversible}+IR$$

Here, *IR* is the product of resistance (*R*) (arising from contact wires, points, and electrolytes) and electric current (*I*). Based on earlier reports [26,27], the catalyst’s performance is evaluated by the overpotential at a current density of 10 mA cm^−2^, which is considered as the standard. The electrocatalysts that can yield a very low overpotential at high current densities are called as ideal electrocatalysts [26].

#### 2.1.2. Faradic Efficiency

It indicates the consumption efficiency of transferable electrons through an external circuit during the course of an electrochemical reaction [28]. Further, it can be determined by the ratio between the experimentally determined and theoretically calculated amount of hydrogen produced as expressed below:

(8) Faradaic Efficiency=H2poducedexperimentalH2producedtheoretical

Usually, the quantity of *H*_2_ produced is experimentally deduced by gas chromatography or water–gas displacement techniques, whereas the theoretical values are obtained by galvanostatic or potentiostatic electrolysis.

#### 2.1.3. Tafel Plot

Tafel slope implies the steady-state current density dependency of the change in overpotential, which can be derived by linear-sweep voltammetry (LSV). Using the Tafel plot for the HER process, the reaction kinetics and reaction mechanism can be revealed using the following expression [29]:

(9) η=b logj+a

In the above equation, *b* indicates the Tafel slope, *j* represents the current density, and *a* is the logarithmic of the exchange current. The Tafel slope is used to determine the reaction kinetics of an electrochemical HER process, and the reaction kinetics reveal the rate determining step, plausible reaction pathway, and fundamental catalytic behavior of the electrocatalysts under equilibrium conditions. Usually, if the value of “*b*” is small, then faster reaction kinetics prevail and vice versa. The Tafel values are always very low with larger current densities for ideal electrocatalysts.

#### 2.1.4. Turnover Frequency

TOF is a facile and direct fundamental activity marker to identifying the efficiency of the catalyst for the electrochemical HER and OER. Furthermore, it is a measure of the quantity of reactant-consumed or product-formed per unit time for the specified catalyst [30]. In other words, it signifies the ratio between the volumetric rate of reaction and the number of active centers per unit volume. Usually, the TOF of a catalyst is reported with the function of overpotential differentiation. A large number of methods are reliable for loading mass with plenty of variations; hence, to rectify such shortcomings, TOF is the most important parameter for determining HER catalytic performance. The exceptional performance of the electrocatalysts can be unveiled by their high TOF values during the electrochemical process in HER. The commonly used equation for calculating the TOF in electrocatalysis is given as follows,

(10) $$TOF=jxN_{A}/\left(Fxnx\tau \right)$$

where *n* indicates the number of electrons necessary to transfer for the production of one molecule of the product, usually for HER and OER, *n* = 2 and 4, respectively. The other parameters *j, F, N_A_,* and *τ* are the current density, Faraday constant, Avogadro constant, and accurate number of active sites of the catalysts during the reaction, respectively.

#### 2.1.5. Gibbs Free Energy (ΔG_H_)

According to the Sabatier principle, the electrical charges could be transferred facilely on the electrocatalyst’s surface, and the bonding of the adsorbed hydrogen yields molecular hydrogen gas as the product. Based on this principle, we can easily determine the Gibbs free energy and reaction kinetics of HER. For instance, if the bonding between the surface of the electrocatalyst and the adsorbed hydrogen is very strong, the desorption reaction is restricted; hence, the Heyrovsky–Tafel step can occur, whereas a weak bonding between the surface of the electrocatalyst and the adsorbed hydrogen adversely affects the Volmer step. The adsorption of hydrogen over the surface of the electrocatalyst is evaluated by the Gibbs free energy function ΔG_H_. The reaction kinetics of the electrochemical process in the HER could be determined from the ΔG_H_ value for hydrogen adsorption. Therefore, ΔG_H_ is one of the important parameters for identifying the electrochemical process in HER.

#### 2.1.6. Electrochemical Active Surface Area

The electrochemical active surface area (ECSA) of an electrocatalyst, especially for a porous substrate, determines the exposure of active sites. This approach is used to identify the surface area with double layer capacitance (EDLC) for the electrochemical process. Therefore, an EDLC within a narrow potential range and at different sweep rates is usually employed. If there is no Faradaic reaction in the measured range, then a substantial differential capacitance equivalent to the EDLC (*C_dl_*) is obtained. Based on the obtained specific capacitance (*C_s_*), the *ECSA* is calculated using the following expression [26,31],

(11) ECSA=CdlCs

According to McCrory et al., the average value of *C_s_* in an alkaline solution is in the range of 40 μF/cm^2^ [32]. If the proposed catalysts have a large number of active exposed sites and highly unsaturated atom co-ordinations, then the *ECSA* is large, indicating the excellent performance of the catalysts.

### 2.2. Necessity of Noble-Metal Free Catalysts for HER

For hydrogen fuel production, it is crucial to develop efficient catalysts and to choose suitable electrolytes to speed up the HER rate. For H_2_ generation, electrocatalytic systems usually integrate expensive noble metals, such as platinum catalysts, because of their unique features, such as low over potentials and ultrafast kinetics, for driving the HERs [33]. For developing electrocatalysts that are highly active, stable, durable, earth-abundant, non-toxic, exceedingly conductive, inexpensive, robust, environment-friendly, and efficacious for next-generation HER non-noble metals are being replaced with commercial high-cost Pt/C because of the scarcity of noble metals [34,35]. Moreover, significant theoretical and experimental research progress has been made by scientists in recent years in this direction. Nevertheless, there is an urgent need to replace the noble metal catalysts with promising highly conductive electrocatalysts that are efficient, cost-effective, and abundant and to achieve the goal of exceptional HER catalytic performance, which is similar to those of the existing electrocatalysts [36].

Designing suitable electrocatalysts and selecting the corresponding electrolytes are two primary influencing factors that accelerate HERs [25]. By employing efficient catalysts for HER, the reaction kinetics can be greatly enhanced by lowering the activation energy, and thus, reducing the overpotential. Many factors should be taken into consideration for developing the ideal electrocatalysts, including the cost of the material, catalytic activity, synthesis method, robustness, and earth-abundant materials with long-term utilization. Electrocatalysts with outstanding kinetics, which show negligible overpotential, are the prerequisite for replacing the expensive and limited Pt-based catalysts in HER. In order to discover the ideal electrocatalyst for HER, significant efforts have been made by researchers all over the world. Exchange current density and Tafel slope are the two key parameters that govern the catalytic efficiency. In particular, inexpensive and abundant electrocatalyst materials are required to replace non-abundant materials like platinum. The HER performance of platinum is superior to any other material, and it performs outstandingly both as an H_2_ evolution and oxidation catalyst with an excellent catalytic activity and is thus the benchmark for HER reactions. Based on previous studies on the development of HER electrocatalysts, it can be suggested that significant progress is being made both experimentally and theoretically in realizing noble-metal-free catalysts [37]. The recent development of TMCs, especially molybdenum and tungsten carbide-based electrocatalysts, for realizing an improved HER activity and their salient catalytic features are summarized in the following sections.

### 2.3. Synthesis of TMCs

**Figure 2:**
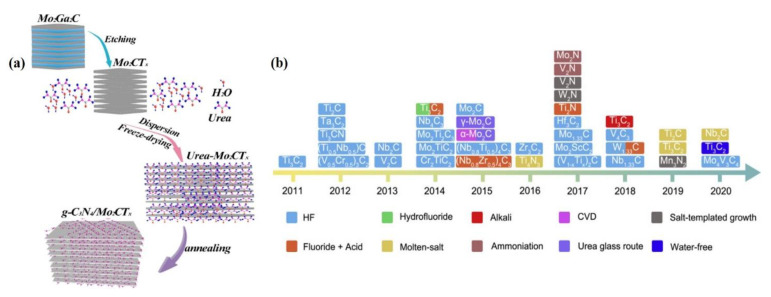
(**a**) Illustration for the preparation of the Mo_2_CT_x_/g-C_3_N_4_ hybrid by mechanical exfoliation using HF as etchant, (**b**) schematic of the synthetic exfoliation protocols of molybdenum and other MXenes reported over the last two decades [14,15]. Copyright 2021 Elsevier.

Usually, there are two different categories of 2D TMCs/TMNs, including non-layered and layered materials. Various synthetic techniques are used to determine the structure of the materials. In order to fabricate 2D TMCs/TMNs, two widely accepted approaches are employed:

(a) The first approach is the top-down approach, which is utilized to construct 2D TMCs/TMNs from their bulk counterparts using liquid exfoliation, mechanical exfoliation, and scotch-tape methods.

(b) In the second bottom-up approach, a 2D nanostructure is formed by chemical vapor deposition (CVD) as well as by solution-related chemical syntheses processes such as hydrothermal and solvothermal and physical vapor deposition processes. Numerous materials synthesized through these processes have their own advantages and disadvantages that depend on the raw materials, processes, cost effectiveness, solvent, and precursors. To date, the top–down process is considered the most effective process to construct MXenes as shown in Figure 2 [15]. Unlike the top–down process, the bottom–up process requires less precursor chemicals and can construct atomic ingredients into a few layers of nanostructures. Further, the bottom-up process comprises several beneficial properties, including morphology control, precise size control, and surface termination of MXenes in a specific ordered manner.

#### 2.3.1. Exfoliation Processes

The exfoliation process is widely used to construct few-layered or single-layered structures of 2D metal carbides from their bulk counterparts. During exfoliation, the type of etching influences the performance of the electrocatalysts. The electrocatalyst materials can be mechanically exfoliated or synthesized by liquid exfoliation via fluoride etching, alkaline etching, electrochemical etching, and water-free etching. For instance, Mo_2_CT_x_ can be prepared liquid exfoliation using different etching solutions like LiF, NaF, KF, and NH_4_F mixed with HCl [38]. In particular, ultrapure Mo_2_CT_x_ can be obtained with a solution mixture of NH_4_ and HCl at the very low temperature of 140 °C. Wan et al. recently constructed a g-C_3_N_4_/Mo_2_CTx hybrid via selective etching with HF, followed by calcination [14]. The schematic illustration of the preparation of Mo_2_CT_x_ from Mo_2_Ga_2_C using HF etchant is shown in Figure 2a. In this case, the ratio of HF and HCl were maintained at 3:2, and the reaction occurred for 72 h. In the second step, urea and the derived Mo_2_CT_x_ were mixed in a ratio of 1:20 and calcined at 550 °C to obtain the Mo_2_CT_x_/g-C_3_N_4_ hybrid. In the case of the scotch-tape process, a piece of tape is adhered to the surface of the bulk 2D material, so that few layers of the top 2D flakes become attached to the tape. Subsequently, this tape is pressed on the preferred substrate to transfer the few-layered 2D flakes. Initially, the scotch tape process (mechanical exfoliation) was employed to synthesize few-layered graphene from bulk graphite. In the recent decade, it has been used to exfoliate 2D MXene materials as well owing to its cost effectiveness and simplicity. Zhang et al. deposited Ti_3_C_2_ thin films over an SiO_2_ substrate by three different processing techniques, including mechanical exfoliation, wafer dipping, and spin coating [39]. Mo_4_Ce_4_Al_7_C_3_ flakes were synthesized through mechanical exfoliation process by Gkountras et al. very recently [40]. The schematic synthetic exfoliation protocols of various molybdenum and other carbide MXenes over last two decades are shown in Figure 2b [15].

#### 2.3.2. CVD

**Figure 3:**
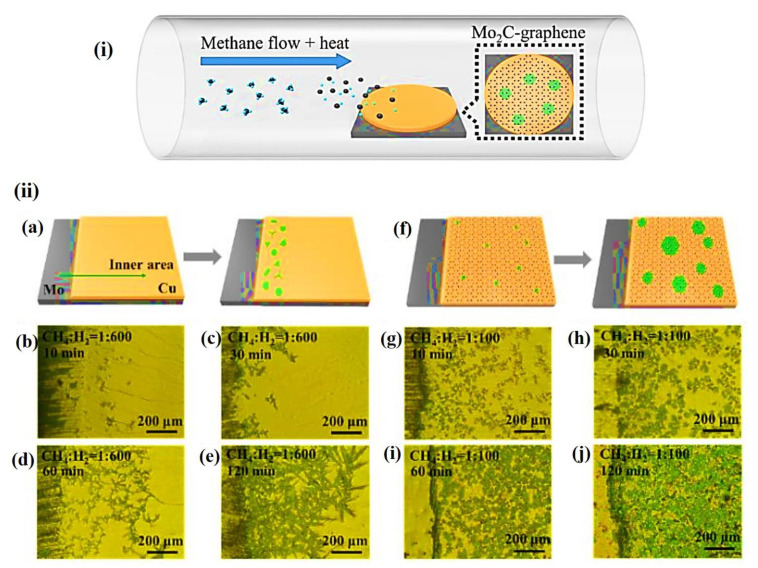
(**a**) Typical representation for the formation of Mo_2_C-graphene over a Cu/Mo substrate under various flow rates (low and high) of CH_4_ to H_2._ (**ii**) (**b**–**e**) Optical microscope image of Mo_2_C over a Cu/Mo substrate under various flow rates (low and high) of CH_4_ to H_2_ at different time intervals of (**b**) 10 min, (**c**) 30 min, (**d**) 60 min, and (**e**) 120 min, (**f**) formation of Mo_2_C-graphene over a Cu/Mo substrate at high flow rate, (**g**–**j**) Optical microscope image of Mo_2_C-graphene over a Cu/Mo substrate under various flow rates (low and high) of CH_4_ to H_2_ at different time intervals of (**g**) 10 min, (**h**) 30 min, (**i**) 60 min, and (**j**) 120 min [16].

The CVD process is a type of bottom–up process to construct highly pure and high-performance solid MXene materials. Usually, a thin layer or a few layers of the required materials are constructed by heating the gaseous reactants in a CVD furnace, between the substrate and gaseous reactants or after the in situ chemical reactions between gaseous precursors. Chuan et al. established the production methodology of 2D ultrathin α-Mo_2_C crystals using CVD to produce a stable few-nanometers-thick layer with a large area [41]. The group used methane as the carbon source and a combination of Cu and Mo foils as the substrate for the growth at a temperature of 1000–1100 °C. Similarly, Geng et al. fabricated Mo_2_C and Mo_2_C-graphene hybrid films of various thickness by CVD using a liquid Cu surface instead of foil substrates [42]. Usage of liquid Cu completely avoids the grain boundary formation as it grows on the supported Mo foil, which in turn acts as a catalyst during the growth process. The same group used methane as the precursor with H_2_, which acts as a reducing as well as carrier gas during the formation at a temperature of 1100 °C. Likewise, Kang et al. recently developed a Mo_2_C-graphene structure over an Mo/Cu substrate by CVD using a different ratio of methane to hydrogen with different time intervals, such as 10, 30, 60, and 120 min, as shown in Figure 3 [16]. Interestingly, the optical images of bare Mo_2_C showed the growth of uneven Mo_2_C crystals at the edges of the substrate (Figure 3b–e). Once graphene is introduced (Figure 3f), it becomes even, and the Mo_2_C-graphene is distributed all over the surface of the substrate as depicted in Figure 3g–j.

Hot filament CVD (HF-CVD) has been used to grow vertically aligned graphene nanoribbons (VA-GNR) from vertically aligned carbon nanotubes (VA-CNTs) [43]. After the growth process, a thin layer of Mo (~75 nm) is deposited over the top of VA-GNR and proceeded to atomic H treated for carburization of metallic Mo. For a competitive performance study, Mo_2_C without a VA-GNR support was prepared. Jia et al. synthesized nanosheets (NSs) of nitrogen-incorporated Mo_2_C (N/Mo_2_C NSs) in a two-step process. First, ultrathin MoO_2_ NSs were prepared by chemical vapor reduction of commercial MoO_3_ powders under an Ar/H_2_ environment at 900 °C for 60 min. In the second step, dicyandiamide (C_2_H_4_N_4_) powder was mixed well with 100 mg of MoO_2_ NSs obtained from the first step and placed inside a tube furnace under an Ar/H_2_ atmosphere. Next, the mixture was subjected to calcination twice simultaneously at 450 °C and 700 °C for 2 h, resulting in the formation of N-Mo_2_C NSs. These synthesized N-Mo_2_C NSs exhibited an excellent HER activity with overpotentials of 48.3 and 99 mV vs. RHE at the cathodic current densities of 1 and 10 mA cm^−2^, respectively. A very low Tafel slope of 44.5 mV dec^−1^, a large exchange current density of 0.1 mA cm^−2^, and excellent long-term stability (stable for more than 12 h) were observed for the resulting catalyst [44].

#### 2.3.3. Hydrothermal/Solvothermal Process

Compared to the CVD process, the hydrothermal and solvothermal processing of 2D TMCs/TMNs facilitates an inexpensive, facile, and large-scale production of these electrocatalysts. Usually, powdery target products can be obtained through the hydrothermal/solvothermal process by adding suitable starting reagents in water/other solvents at a high pressure and temperature. Compared to the hydrothermal approach, other solvents are used to replace water in the solvothermal method. Notably, the solvothermal reaction temperature should be higher than the boiling temperature of the solvent when all the starting reagents are kept in a closed vessel. Some interesting reports on hydrothermally processed 2D TMCs/TMNs are discussed herein to gain more insights into these materials and their potential as electrocatalysts. Glucose hydrolyzed Mo_2_C-RGO (RGO denoted reduced graphene oxide) hybrids were developed by Pan et al. by hydrothermal carbonization using a graphene oxide (GO) suspension and ammonium heptamolybdate at 180 °C [45]. Interestingly, amorphous Mo_2_C changed into 3D particles with an average size of 50 nm in the absence of GO sheets. Cheng et al. synthesized β-Mo_2_C with a Mo:amine ratio of 1:4 (heptamolybdate ((NH_4_)_6_Mo7O_24_·4H_2_O) or sodium molybdate (Na_2_MoO_4_) in 4-Cl-ophenylenediamine in 60 mL of deionized water) in an acidic (pH 3) solution to ensure the formation of proper amine–oxide hybrids [46]. In the case of Fe-doped molybdenum carbide, 0.02–0.1 equimolar ratio of FeCl_3_·6H_2_O was added to a wet precipitate, and a usual purification criterion was followed. The synthesized β-Mo_2_C was further subjected to a long-term stability analysis at both low (−140 mV) and high (−240 mV) overpotentials for an appropriate period of 9 h. In contrast, two-step protocols were used by Meihong et al. to synthesis Mo_2_C/CC (CC represents carbon cloth) [47]. In the first step, a hybrid between the molybdate anions and the cetyltrimethyl ammonium cations was formed over the CC by hydrothermal processing. Next, a simple in situ thermal treatment was performed in an Ar atmosphere to form Mo_2_C@C hybrids at 900 °C. The resulting Mo_2_C@CC exhibited a record high HER activity of 140 mV @ 10 mA cm^−2^ and 124 mV per dec^−1^ (overpotentials and Tafel slope, respectively).

## 3. HER Activity of 2D TMCs

### 3.1. Molybdenum Carbide (Mo_2_C) and Its Composite

Molybdenum carbide Mo_x_C is known to exhibit high electronic conductivity and has a Pt-like electronic configuration. The excellent HER activity of Mo_2_C in both alkaline and acidic media was first demonstrated in 2012 [48]. Nevertheless, the application of Mo_2_C as a substitute of noble metal Pt is hindered by two notable obstructions [19,22]. First, the temperature-controlled reaction or commercial carburization in gas phase restricts the nanostructuring. Second, the reduction of metal starting reagents needs a high elevated temperature and highly flammable hydrocarbon gases such as CO, CH_4_, and C_2_H_6_, which damage the nanostructure, reduce the number of active sites, and deteriorate the reaction surface. Hence, engineering the gas phase constituents, surface area is essential to forming various phases of metal carbides with various catalytic activities. The ultra-prime progress on synthesis protocols has been made to overcome these problems. To avoid particle coking and sintering, various process like incorporation of carbon supports or metal–organic frameworks (MOFs) into the carbide matrix are adopted. Further, the inclusion of heterometal atoms also improves the overall catalytic activity. These issues are discussed in detail in the following sections with examples.

#### 3.1.1. Molybdenum Carbide (Mo_2_C)

Initially, molybdenum carbide (Mo_2_C) was prepared by Qamar and his colleague by a two-step fabrication process that involved the incorporation of an Mo source into the MOF matrix MIL-53(Al). Next, carburization was conducted to facilitate the growth and nucleation of Mo_2_C nanocrystals into a well-defined porous texture. Moreover, they obtained an overpotential of 165 @ 10 mA, a low Tafel slope of 63.6 mV dec^−1^, and observed a prolonged durability (20 h) in 1.0 M KOH medium [49].

By contrast, Chen et al. prepared β-Mo_2_C nanoparticles (NPs) by an in situ carburization using CNTs and ammonium molybdate. These NPs exhibited much smaller onset potential values of 63 @ 1 mA cm^−2^ and 105 @ 10 mA cm^−2^, a small Tafel slope of 55.2 V dec^−1^, and an exchange current density of 1.4 × 10^−2^ mA cm^−2^ [50]. The β-Mo_2_C solution was prepared by mixing ammonium heptamolybdate ((NH_4_)_6_Mo7O_24_·4H_2_O) or sodium molybdate (Na_2_MoO_4_) in 4-Cl-ophenylenediamine with an Mo:amine ratio = 1:4. Acidic pH was maintained by adding hydrochloric acid until the formation of amine–oxide hybrid precipitates. Pure β-Mo_2_C was obtained as a result of centrifugation at 5500 rpm for 10 min; the solution was heated to 50 °C with stirring for 2.5 h. In contrast, for Fe-doped molybdenum carbide synthesis, 0.02–0.1 molar equivalents of FeCl_3_/6H_2_O were added in 60 mL of water. Thus, the prepared β-Mo_2_C exhibited long-term durability at both lower and higher overpotentials of −140 and −240 mV, respectively, for 9 h. Later, Jia et al. reported nitrogen-doped Mo_2_C NSs prepared using a two-step process. These NSs exhibited an excellent HER activity with an overpotential of 48.3 and 99 mV vs. RHE at cathodic current densities of 1 and 10 mA cm^−2^, respectively. A very low Tafel slope of 44.5 mV dec^−1^, a large exchange current density of 0.1 mA cm^−2^, and excellent long-term stability of more than 12 h were observed [44]. Chaoyun et al. developed Mo_2_C by a combined hydrothermal and calcination process. The product obtained from the hydrothermal treatment was heated in a tube furnace at 800 °C for about 3 h in an Ar atmosphere. Finally, β-black molybdenum carbide (β-Mo_2_C) was obtained by H_2_ reduction for 20 min. The as-prepared catalyst exhibited the best HER activity with the smallest onset potential of 80 mV @ 1 mA cm^−2^, a small Tafel slope of 55 mV dec^−1^, an overpotential of −165 mV @ 10 mA cm^−2^, and a large current density of 60 mA cm^−2^ @ 200 mV [51].

Chen et al. prepared multiple phases of molybdenum carbide (Mo_2_C) by solution-based processes. The amine–metal oxide hybrid was precipitated from an aqueous solution of ammonium molybdate and 4-Cl-o-phenylenediamine (4Cl-oPDA) by adjusting the pH to below 3. The amine–metal oxide hybrid was heated at 750 °C for 12 h to form α-MoC_1−x_, at 850 °C for 12 h to produce β-Mo_2_C, and at 850 °C for 24 h to form γ-MoC. p-Phenylenediamine (pPDA) was used as the precursor to produce η-MoC at 1050 °C with a dwell time of zero. The β-Mo_2_C electrode exhibited a Tafel slope of 120 mV/decade and an exchange current of 17.29 μA cm^−2^ (*η* = 180–220 mV). Comparatively, the Tafel analysis of γ-MoC yielded an exchange current of 3.2 μA cm^−2^ and a Tafel slope of 121.6 mV dec^−1^ (*η* = 270–310 mV) [46].

The MoC-Mo_2_C hetero-nanowires (NWs) were prepared by immersing (NH_4_)_6_Mo_7_O_24_·4H_2_O in aniline solution by Huanlei et al. During the preparation process, it is keen to note that the solution should be kept at 50 °C for 4 h in an oil bath for its slower reaction process; the final products were filtered and thoroughly washed with ethanol, and then dried at 50 °C overnight. HER parameters such as overpotential, Tafel slope, and onset potentials in 0.5 M H_2_SO_4_ and 1.0 M KOH were 126 and 120 mV, 43 and 42 mV dec^−1^, and 38 and 33 mV, respectively. The exchange current density (j_0_) of MoC was 1.1 × 10^−2^ mA cm^−2^ [52].

Pan et al. successfully synthesized novel molybdenum carbide (Mo_x_C) using a simple one-pot pyrolysis method. One-dimensional MoO_3_-EDA prepared by the already reported procedure was subjected to a pyrolysis treatment at 600 °C under an Ar gas atmosphere, and molybdenum carbides Mo_x_C with high-valence state Mo nanobelts were obtained. The as-prepared products displayed tremendous HER electrocatalytic activity with a low onset overpotential of 50 mV and a small Tafel slope of 49.6 mV dec^−1^ in an acidic medium (0.5 M H_2_SO_4_). In addition, the catalysts necessitate merely overpotentials of 143 and 234 mV to achieve current densities of 10 and 220 mA cm^−2^, respectively with good durability after 2000 cycles [53].

Bin et al. presented a unique synthesis strategy to produce porous MoC_x_ nano-octahedrons via an in situ wet chemical process and confined carburization reactions. The group used metal–organic frameworks (MOFs) and guest polyoxometalates (POMs) that resides inside the pores of MOFs and acts as receiver of the guest metal species into the MOFs. The co-precursor—namely transition metals (Mo, W, and V)—carbides enable easy synthesis in the presence of metal species that would be impossible if single MOF sources were used. MoC_x_ nano-octahedrons possess HER onset potentials of 87 and 92 @ 1 mV, 142 and 157 @ 10 mV vs. RHE, low Tafel slopes of 53 and 59 mV dec^−1^, and exchange-current densities of 0.023 and 0.029 mA cm^−2^ in acidic and alkaline media with excellent long-term stability (more than 10 h) [54]. A meso-microporous structured 3DHP-Mo_2_C catalyst fabricated by Meng et al. involves a four-step process. The constructed 3DHP-Mo_2_C apparently exhibited a comparatively larger current density—1 mA cm^−2^ with a low overpotential of 75 mV—than 3DHPC and Com-Mo_2_C (235 and 202 mV at the same current density). Further, 3DHP-Mo_2_C exhibits a relatively low Tafel slope value with excellent stability (10 h) in 1M KOH solution [55].

#### 3.1.2. Mo_2_C and Carbon Composites

For electronic modification and nanostructuring, different carbon-based supports including GO, CNTs, and carbon fiber are employed along with metal carbides. The inclusion of well-defined carbon supports enhances the particle dispersity and so the number of active sites, thereby leading to improvement of the electronic conductivity and overall catalytic performance towards HER activity by means of altering the electrochemical structure. A various carbon-supported metal carbides for improved HER performance are elaborated in this section.

Through a solid-state process, Cui et al. decorated Mo_2_C NPs over graphitic carbon sheets (Mo_2_C/GCSs) using (NH_4_)_6_Mo_7_O_24_·4H_2_O and sodium alginate (ALG) as the precursors at 900 °C under an Ar atmosphere. The resulting Mo_2_C/GCSs catalyst was tested in 0.5 M H_2_SO_4_ for HER, and a low overpotential (120 mV @ 1 mV) and Tafel slope (62.6 mV dec^−1^) were obtained in the measured range. In addition, the catalyst showed good electrical conductivity with a large exchange current density of 0.125 mA cm^−2^ and long durability over 3000 electrochemical cycles.

Using a similar solid-state strategy, Pu et al. developed Mo_2_C quantum dot-based nitrogen-incorporated graphitic carbon layer (Mo_2_C-QDs/NGCLs). Firstly, through the sonication method, the precursors such as chitosan biopolymer and ammonium molybdate ((NH_4_)_6_Mo_7_O_24_·4H_2_O) were dissolved in distilled water. During this stage, a chitosan–Mo complex could be formed by means of MoO_4_^2−^ conjugate with OH and NH_2_ of chitosan. Later, the formed complex was subjected into an annealing process at 900 °C under Ar environment for 2 h, producing a hybrid Mo_2_C-QDs/NGCLs material. For HER analysis, Mo_2_C-QDs/NGCLs delivers the appropriate overpotential of 150 mV @ 10 mA cm^−2^ and very mere Tafel slopes values of 81.9 mV dec^−1^ in 0.5 M H_2_SO_4_ suggesting its good stability. The same catalyst offers the low overpotential and Tafel slope values of 111 mV and 57.8 mV dec^−1^ @ 10 mA cm^−2^ even in the exceptional alkaline 1.0 M KOH electrolyte [56].

Huang Yang’s group developed Mo_2_C NPs using a widely used two-step strategy using ammonium molybdate tetrahydrate ((NH_4_)_6_Mo_7_O_24_·4H_2_O) and dopamine hydrochloride (C_8_H_11_NO_2_·HCl)) as the precursors. The uniform dispersion of Mo_2_C over carbon micro flowers (Mo_2_C/NCF) was realized through the self-polymerization of dopamine. For the HER properties, the overpotentials of 85 and 144 mV @ current densities of 1 and 10 mA cm^−2^, respectively, for Mo_2_C/NCF were obtained. Tafel study divulged that the linear region exhibited a slope of 55 mV decade^−1^ in a 0.5M H_2_SO_4_ solution. HER activities of Mo_2_C/NCF in 1 M KOH were found to be enhanced with an overpotential of 38 and 100 mV @ 1 and 10 mA cm^−2^, respectively, and an excellent stability (8 h) [57].

Dezhi et al. constructed Mo_2_C NPs embedded in Ketjenblack carbon (KB) by a simple in situ carbonization process. The augmented Mo_2_C/KB catalyst reveals a distinctive HER activity in both acidic and alkaline conditions with the least Tafel slopes of 49 and 48 mV dec^−1^, and overpotential of 180 mV @ 10 mA/cm^2^, an exchange current density of 3 × 10^−3^ mA cm^−2^, and an excellent stability (10 h) [58]. Can et al. prepared hierarchical Mo_2_C/C NS hybrids by employing a novel preparation process involving the carburizing of ammonium molybdate and citric-acid-coated NaCl cube crystals under an Ar gas atmosphere. HER performance of Mo_2_C/C hybrids is studied at both alkaline and acid media with the very low overpotential of 125 mV @ 10 mA cm^−2^ in 1 M KOH and 180 mV @ 10 mA cm^−2^ in 0.5M H_2_SO_4_, while the observed Tafel values are 72 mV dec^−1^ in 1.0 M KOH and 71 mV dec^−1^ in 0.5M H_2_SO_4_ [59].

Cuncai et al. developed MoC/C by a simple one-step ultrasonic spray pyrolysis (USP) by dissolving MoCl_5_ in ethanol. During the USP process, the starting material passes via the heating zone inside the series outlets to safeguard the indistinguishable heating. The resultant MoC/C from the pyrolysis has offered the least Tafel slope of 63.6 mV dec^−1^ @ 20 mA cm^−2^ in 0.5 M H_2_SO_4_, and exists a long term stability during the electrolysis [60]. Chunyong and coworkers synthesized nanostructured molybdenum carbide of smaller size ranging from 2.5 nm and 5 nm for MoC and Mo_2_C on a graphene support, respectively, through a facile in situ approach. For the production of Mo_2_C-G and MoC-G through this approach, GO powders and (NH_4_)6Mo_7_O_24_·4H_2_O were employed as the starting reagents. The HER performances of Mo_2_C-G and MoC-G with the least overpotential (*η*_10_) of 150 and 221 mV @ 10 mA cm^−2^, values of Tafel slopes of 57, and 88 mV dec^−1^ respectively. The exchange current density (j_0_) of the MoC-G and Mo_2_C-G are observed to be 0.00255 mA cm^−2^ and 0.00258 mA cm^−2^ respectively and demonstrates the exceptional stability over 20 h [61].

**Figure 4:**
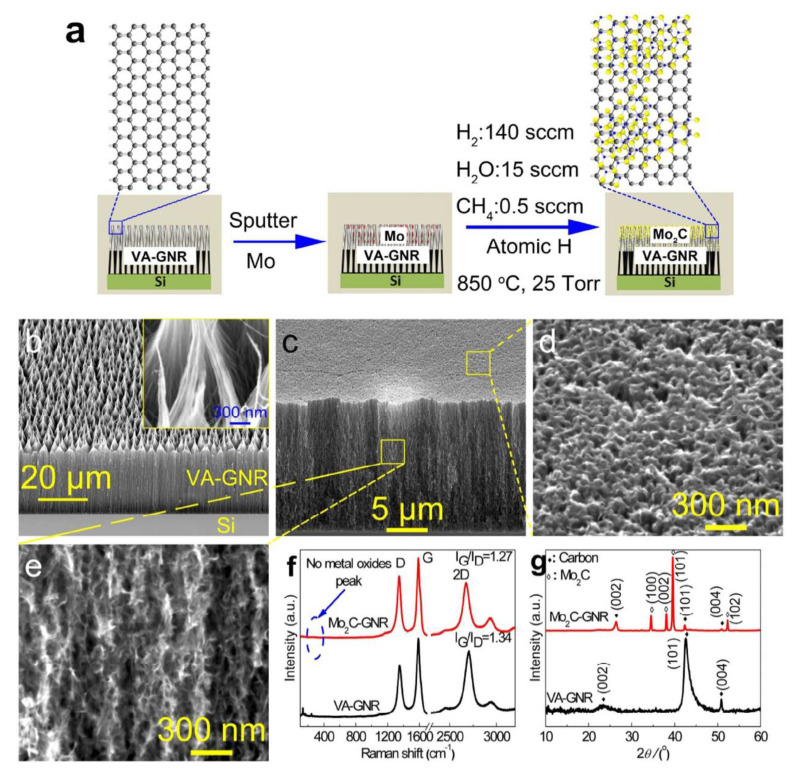
(**a**) Schematic showing the formation of Mo_2_C over VA-GNR, (**b**–**e**) field-emission SEM image of VA-GNR and Mo_2_C-GNR at high and low magnifications, (**f**,**g**) their corresponding Raman spectra in the range of 300–3100 cm^−1^ and XRD patterns in the 2θ range of 10–60°. These spectral analyses confirmed the formation of Mo_2_C over the GNR matrix [43]. Copyright 2017 The American Chemical Society.

Huo Lili and his team established an inexpensive, noble-metal-free mesoporous Mo_2_C/graphene (m-Mo_2_C/G) catalyst with two-dimensional layered structure by a nanocasting method using glucose as a carbon source for in situ assembled mesoporous KIT-6/graphene (KIT-6/G) as a template. The m-Mo_2_C/G catalyst outrages large catalytic behavior with outstanding long-term stability for HER over a wide pH range at small onset potential of 8 mV. Overpotential (*η*_10_) for a cathodic current density of 10 mA cm^−2^ of 135 mV, a Tafel slope of 58 mV dec^−1^, and an exchange current density of 6.31 × 10^−2^ mA cm^−2^ in acidic media have been observed. Similarly for alkaline medium, an onset potential of 41 mV, η_10_ of 128 mV, an exchange current density of 0.00409 mA cm^−2^, and Tafel slope of 56 mV dec^−1^ have been observed [8]. By using the HF-CVD process, vertically aligned graphene nanoribbons (VA-GNR) were grown from vertically aligned carbon nanotubes (VA-CNTs) [43]. After the growth process, a thin layer of Mo (~75 nm) was deposited on the top of VA-GNR and atomic H treatment was conducted for carburization of metallic Mo as displayed in Figure 4. For a comparison, the performance study of Mo_2_C without the VA-GNR support was also prepared.

The corresponding scanning electron microscopy (SEM) images reveal the vertically aligned nanoribbons over Mo_2_C as shown in Figure 4b–e. Its respective spectral analyses such as Raman and X-ray diffraction (XRD) imply the purity of the prepared catalyst and confirms the absence of metal oxide in the prepared materials as shown in Figure 4f,g.

**Figure 5:**
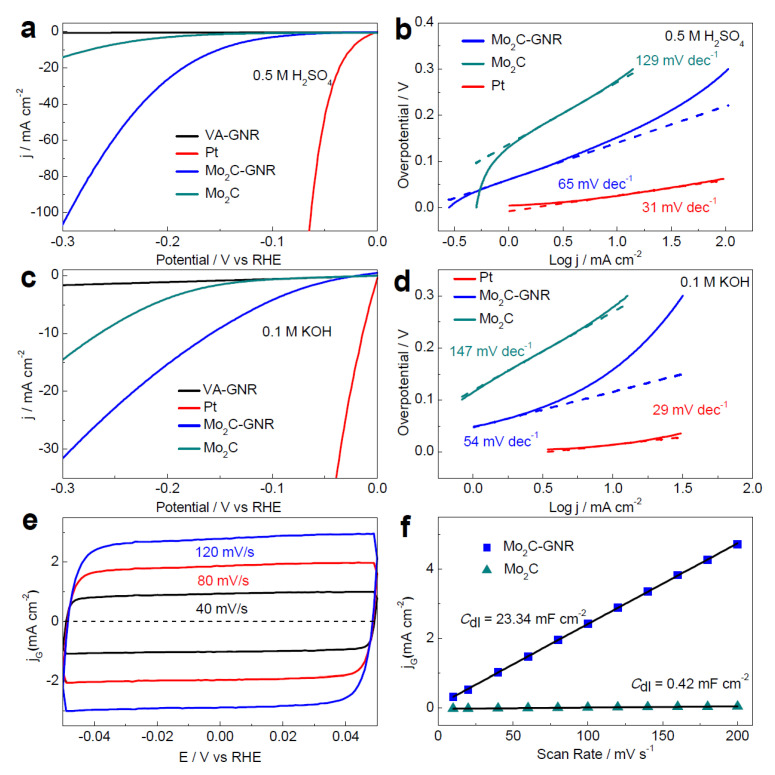
(**a**,**b**) LSV and Tafel slope plots of Mo_2_C nanocrystals over VA-GNR, bare VA-GNR and Mo_2_C in an acidic medium, (**c**,**d**) LSV and Tafel slope plots of Mo_2_C nanocrystals over VA-GNR, bare VA-GNR, and Mo_2_C in an alkaline medium, (**e**,**f**) corresponding CV and current density vs. scan rate plots at various scan rates to determine *C_dl_* [43]. Copyright 2017 The American Chemical Society.

As depicted in Figure 5a–d, the HER performance of Mo_2_C-GNR in acidic (0.5 M H_2_SO_4_) as well as alkaline (1 M KOH) solutions confirmed the excellent HER activity of Mo_2_C-GNR. Compared to the current density values (29 and 31 mV) of Pt/C and those of the stand-alone Mo_2_C (~275 and ~266 mV), the above catalyst Mo_2_C-GNR exhibited a better current density of 10 mA cm^−2^ at a low (ƞ) of ~152 and ~121 mV. Similarly, the Tafel slope values of the Mo_2_C-GNR and Mo_2_C catalysts in acidic and alkaline solutions were 65 and 54 mV dec ^−1^ and 129 and 147 mV dec^−1^, respectively.

As shown in Figure 5e, a systematic cyclic voltammetry (CV) test was conducted to determine the EDLC behavior (C_dl_) of the resultant catalysts. As evident from Figure 5f, the capacitance values of Mo_2_C-GNR and Mo_2_C electrodes are 23.34 and 0.42 mF cm^−2^, along with the roughness factors of 1060 and 19, respectively.

Kasinath et al. synthesized RGO-based composites with Mo_2_C rods using a two-step process that involved aniline complexation followed by annealing. The RGO-Mo_2_C catalyst exhibited an excellent electrochemical activity toward HER in an acidic medium and achieved the highest current density of 125 mA/cm^2^ at 400 mV and the lowest Tafel slope of 67 mV dec^−1^ [62]. The presence of GO in the Mo_2_C-RGO composite reduced the particle size (~10 nm) and chiefly improved the carbonization reaction. Kai et al. fabricated Mo_2_C-NCNTs by a two-step process. The Mo_2_C-NCNTs offers the lowest overpotentials of 72 and 147 mV @ 1 and 10 mA cm^−2^ current densities, respectively. The Mo_2_C-NCNTs catalyst has further shown the least Tafel slope of 71 mV dec^−1^, exchange current density of 114.6 μA cm^−2^ [63]. Youn et al. fabricated Mo_2_C/CNT-graphene using CNTs and graphene oxide (GO) in ethanol solution followed by the addition of metal precursor (MoCl_5_) resulting in the Mo orthoester formation. On addition of urea (1 molar ratio (R)) yielded a metal-urea complex on CNT-GO, which upon heat treatment under N_2_ atmosphere at 750 °C results in Mo_2_N/CNT-GR hybrids. Upon increasing, the R-value by a factor of eight, Mo_2_C/CNT-GR been generated instead of Mo_2_N. The unreacted nitrogen and oxygen atoms were removed from the precursor solutions as NOx during the heat treatment. The Mo_2_C/CNT-graphene displays the maximum activity for HER with a least overpotential and Tafel slope of 62 mV and 58 mV dec^−1^ respectively @ 1 mA cm^−2^. The calculated exchange current density is in the range of 6.20 × 10^−2^ mA cm^−2^ along with very good stability in H_2_SO_4_ solution [64].

Jian et al. prepared Mo oxyanions-loaded cotton T-shirts using molybdenum carbide NP (BCF/Mo_2_C) electrodes via a direct annealing treatment method. The BCF/Mo_2_C displayed an enhanced electrocatalytic performance with extremely low overpotentials of 88 and 115 mV to drive a current density of 20 mA cm^−2^, and Tafel slopes of 84.8 mV dec^−1^ and 52.4 mV dec^−1^ in alkaline and acidic media. The hybrid’s 50 h stability test showed a decreased cathodic current density in both the alkaline and acidic solutions [65]. Mo_2_C-based crosslinked carbon networks (Mo_2_C/CLCN) were prepared by Jia et al. Equal volumes of CuCl_2_·2H_2_O (1.4 mmol) and (NH_4_)_6_Mo_7_O_24_·4H_2_O (1 mmol) solutions were mixed subsequently and heated at 450 °C for 4 h under an Ar-H_2_ (10%) atmosphere. After the calcination, the black Cu-MoO_2_ rods were obtained. Furthermore, Cu-MoO_2_ rods has been heated up to 1000 °C under Ar (10 sccm) atmosphere to form Mo_2_C based crosslinked carbon network (Mo_2_C/CLCN). Mo_2_C/CLCN display a low onset potential of −85 mV @ 10 mA cm^−2^ an operating overpotential of 145 mV @ 10 mA cm^−2^ with a Tafel slope of 48.2 mV dec^−1^, with a large exchange current density (0.062 mA cm^−2^), and outstanding long-term cycling stability in acidic electrolyte [66].

Fan et al. prepared mesoporous Mo_2_C/nanocrystals by dispersing ammonium molybdate (0.225 g) in ethylenediamine (1.35 g) by ultrasonication and stirring for at least 20 min. Then, carbon tetrachloride (2.31 g) was added, and the resultant mixture was heated, refluxed, and stirred at 90 °C for 6 h. Next, heat-treatment of the polymer composites was performed under nitrogen flow at 800 °C with a heating rate of 3.08 °C min^−1^ and kept under these conditions for 6 h to carbonize the polymer. The optimized electrocatalyst Mo_2_C/nanocrystals requires relatively low overpotentials of 140 mV to produce a current density of 10 mA cm^−2^ for the electrocatalytic HER, a Tafel slope of 116 mV dec^−1^, a large exchange current density of 0.0361 mA cm^−2^ and to remain stable for 120 h in acidic medium [67].

#### 3.1.3. Heterometal Atom Doped Mo_2_C

The doping of hetero-metal atoms with metal carbides especially molybdenum carbide could effectively alter the inherent electrocatalytic activity of Mo by changing its electronic structure. Since, the d-band electron structure of Mo_x_C is same as Pt, the Mo has the restricted activity due to their empty d-orbitals which in turns to cause strong hydrogen-binding and so lower the rate of hydrogen desorption. The doping of hetero-metal atoms in Mo_x_C could downshift the d-band electron center regarding to EF which enhances the kinetic of HER activity. For instance, Ji-Sen et al. designed and fabricated a 2D hybrid consisting of Mo_2_C incorporated by N, P-codoped carbon shells and RGO (Mo_2_C@NPC/NPRGO) by following a three-step protocol. Mo_2_C@NPC/NPRGO has established outstanding HER performance in both alkaline and acidic media with a very low overpotential (34 mV for 10 mA cm^−2^), exchange current density 1.09 mA cm^−2^, Tafel slope (33 mV dec^−1^), and long stability for 10 h in 0.5 M H_2_SO_4_ [68]. Jun et al. constructed Mo_2_C porous nanostructures in a controlled way, including 2D NSs and 1D NWs. First, the MoO_3_ NSs were prepared by hydrothermal processing. In this process, the precursor solutions, containing a mixture of 2-methylimidazole and cobalt nitrate hexahydrate, were immersed into methanol. This led to the formation of MoO_3_@ZIF-67 hybrid NSs as the ZIF-67 layer was coated on top of the MoO NSs. The coated thin layers were subjected to carburization process under ultrapure Ar atmosphere at 800 °C for 3 h to form nitrogen- and cobalt-doped Mo_2_C porous NSs (2D-N, Co-Mo_2_C), which largely contract the morphology of the MoO_3_ NSs. 2D-N, Co-Mo_2_C shows an onset potential as low as 25 mV @ 1 mA cm^−2^, 71 mV @ 10 mA cm^−2^. Tafel slope of 2D-N, Co-Mo_2_C is 40 mV dec^−1^ in 0.1 M HClO_4_. 2D-N, Co-Mo_2_C shows an onset potential as low as 36 mV @ 1 mA cm^−2^, 92 mV @ 10 mA cm^−2^, Tafel slope of 2D-N, Co-Mo_2_C is 47 mV dec^−1^ in 0.1 M KOH [69].

Co-doped Co-Mo_2_C NWs were prepared by Huanlei et al. using Mo_3_O_10_(C_6_H_5_NH_3_)_2_·2H_2_O and a varied CoCl_2_⋅6H_2_O precursors in aqueous aniline solution. 1 m HCl aqueous solution was added to the aniline solution to adjust the pH level to 4.5 for thermodynamic reaction. Finally, the pink products obtained were filtered using ethanol, and then dried at 50 °C overnight and then calcined at 750 °C for 5 h resulting in the formation of Co-doped Co-Mo_2_C NWs. Before testing for HER activity, the products were treated through 1.0 m HCl to remove residual Co species. For preparing Mo_2_C NWs, the similar procedures were employed without the addition of CoCl_2_⋅6H_2_O. Co-Mo_2_C NWs (Co/Mo ratio—0.02) has exhibited a low overpotential (*η*_10_ = 140 and 118 mV @ 10 mA cm^−2^; η_100_ = 200 and 195 mV @ 100 mA cm^−2^), least Tafel slope values (39 and 44 mV dec^−1^), and merely lower onset overpotential (40 and 25 mV) in 0.5 m H_2_SO_4_ and 1.0 m KOH, respectively [70]. Through a joined hydrothermal and carburization process, Ni-doped Mo_2_C over nickel foam (Ni-Mo_2_C/NF) has prepared by Xiong et al. [71]. The resultant Ni-Mo_2_C/NF is observed to be extremely active toward HER and needs a low onset potential of 21 mV @ 1 mA/cm^2^, 150 mV @ 100 mA/cm^2^ which are noticeably smaller than the onset potential of bare Mo_2_C/NF (87 mV) @ 1 mA/cm^2^. The calculated Tafel slopes of 54.2 and 36.8 mV dec^−1^ are found for bare Mo_2_C/NF and Ni-Mo_2_C/NF respectively. Further Ni-Mo_2_C/NF delivers the exchange current density of 0.51 mA cm^−2^ which is 12 folds greater than that of Mo_2_C/NF. Likewise, the Mo_2_C@nanocrystals catalyst has fabricated by Yipu et al. provides a mere low Tafel slope value of 60 mV dec^−1^ and a maximum exchange current density of 0.096 mA cm^−2^ with excellent stability in HER even after 80 h-long over a wide pH range (pH 0–14) [72].

Shiping et al. employed solid-state thermolysis reactions to prepare molybdenum-carbide-modified N-doped carbon-vesicle encapsulated Ni NPs (Mo_x_C-Ni@ NCV) Mo_x_C-Ni@ NCV which revealed high catalytic HER activity in acidic electrolyte, indicating a synergistic effect between γ-MoC and Ni. The HER catalyzed by sample Mo_x_C-Ni@ NCV has reached the stable state with the least overpotential and Tafel slope of 75 mV and 45 mV dec^−1^ respectively @ 10 mA cm^−2^. The same catalyst delivers the large exchange current density of 0.95 m A cm^−2^ in the measured range [73]. A hexamethaylenetetramine (HMT) based one-step thermal decomposition method was employed by Yuan et al. to develop Co_3_Mo_3_C decorated CNT (Co_3_Mo_3_C/CNT). The purpose of adding HMT favors the formation of molybdate ion ligand, besides acting as self-reducing agent. Co_3_Mo_3_C/CNT electrocatalyst, points the low onset and overpotentials of 42 mV, and 124 mV, exchange current density of 0.415 mA cm^−2^ and Tafel slope of 249 mV dec^−1^ @ 10 mA cm^−2^ in seawater [74].

Cuicui et al. used in situ confining carburization to prepare well-regulated structures of Mo_2_C NSs in N-doped carbon (MCNS/NC) using Mo-based inorganic–organic lamellar mesostructures at 900 °C. They demonstrated the HER efficiency at the least overpotential of 19 mV and an onset-potential of ~0 mV @ 10 mA cm^−2^. The determined Tafel slope was 28.9 mV dec^−1^ in acid media [75]. Zhao et al. performed a two-step process to fabricate MoS_2_/Mo_2_C hybrid NSs. Firstly, they vertically aligned MoS_2_ has grown over carbon fiber paper (CFP) by hydrothermal method (200 °C using Na_2_MoO_4_ and thiourea). Then, the formed MoS_2_ NSs are undergone carburization in Ar/H_2_/CH_4_ atmospheres at 750 °C to form MoS_2_/Mo_2_C hybrid NSs. The as-prepared hybrid MoS_2_/Mo_2_C NSs shows the least Tafel slope of 48 mV dec^−1^ with an outstanding HER performance (63 mV @ 10 mA/cm^2^) as well as excellent stability [76]. The electrochemical characteristics of various Mo_2_C-based electrocatalysts for improved HER activity are listed in Table 1.

It is well known that TMCs have no limits to control the carbon content in TMCs’ phase and so no forfeits for the position of the active sites. In specific the carbon atoms in the metal carbides like Mo_2_C and WC are occupied at the interstitial site of metal ion (Mo and W) mostly stimulates the mixing and re-hybridization processes between the d-orbitals of metal atoms and s, p orbital of C-atom [77]. Henceforth, the electronic density of d-band at the Fermi level matches almost that of Pt-atom and showed Pt-like catalytic behavior. Additionally, as stated above, to obtain the thorough insight into the metal carbides catalysts such as Mo_2_C and WC active sites towards HER, the ECSA is one of the dominant factors to determine the activity of the catalyst which is in linear-dependent with the C_dl_ of the resultant catalysts. In addition, to identify the real accurate active sites of the catalysts, the Gibbs free energy |Δ*G*_H*_| could be calculated for all sites (including Mo and C in Mo_2_C; W and C in W_2_C or WC) [77].

According to various reported literatures, the |Δ*G*_H*_| values at various adsorption sites of Mo is lower than the carbon sites suggesting the actual active site is Mo in Mo_2_C catalyst where the hydrogen is favorably profoundly adsorbed or desorbed [77,78]. Similarly, the real active site W in WC catalyst could be identified by its low |Δ*G*_H*_| at various W-adsorption sites and so prefers hydrogen adsorption/desorption. Mostly active edge sites have contributed for the efficient catalytic activity of metal carbides including Mo_2_C and W_2_C/WC. In the specialized case, few research groups purposefully activated the basel or the planar edge sites to enhance the overall catalytic activity of metal carbides [2,79,80]. Generally, to confirm the existence of active sites and their roles towards HER process, various techniques including X-ray absorption near edge structure analysis, DFT calculations, and Fourier transform of the Mo or W extended X-ray absorption structure analysis can be used [81].

### 3.2. Tungsten Carbide (WC/W_2_C)

Tungsten carbide was first invented by Levy and Boudart in the year 1973. This material displays Pt-like performance in various catalytic reactions [82]. In addition, tungsten carbide shows a Pt-like performance with high immunity to catalytic poisonous gases such as CO and H_2_S; such a feature is not observed even in Pt. Based on the typical metal-carbon bonding, tungsten carbide is analogous to molybdenum carbide and has progressed in a parallel technique. There are two different phases of tungsten carbide including WC and W_2_C, which are commonly investigated as the electrocatalysts for HER process [82]. As compared to WC, W_2_C possesses higher catalytic activity [83]. Since, the ratio of tungsten to carbon starting reagents in W_2_C is uncontrollable, commercial carbide synthesis protocols using hydrocarbon gases like CO, C_2_H_6_ and CH_4_ could not work. This may be attributed to the extremely fast carbon diffusion at the solid–gas interface. However, this phase of tungsten carbide could be achieved by using crystalline non-volatile solid carbon precursors such as multi-walled carbon nanotube (MWCNTs), RGO, graphene, etc. At that time, various attempts had been made to determine the viability of employing tungsten carbide and its compounds as electrocatalysts for water splitting reactions. The nanocrystalline thin films based on tungsten carbide has showed excellent catalytic activity towards HER and reported elsewhere by Zheng et al. [84].

#### 3.2.1. Various Nanostructures of Tungsten Carbide Catalyst

The 1D tungsten carbide (WC) NWs with high porosity are initially established by Ren Bowen and his co-worker by two-step synthetic protocols involving solution treatment followed by carbonization process. The as-prepared catalyst displays a very good HER catalytic activity with the least onset potential of 39 mV. In acidic medium, the calculated overpotential *η*_10_ and Tafel slope of 118 mV and 55 mV dec^−1^ @ 10 mA cm^−2^. Whereas in alkaline medium, same catalyst delivers the onset potential, *η*_10_, and Tafel slopes of 56 mV, 122 mV, and 56 mV dec^−1^ respectively indicating its excellent catalytic activity [85]. Likewise, another 1D form of nanocrystalline WC nanowalls were prepared by Jin and his group through a two-step CVD processes. Initially the nanocrystalline diamond layer was fabricated over silicon substrate by HF-CVD process. Later, the WC nanowall was grown over the NCD layer through the direct current plasma CVD process. The prepared catalysts reveals excellent HER performance with 248mV of overpotential, and 67 mV dec^−1^ of Tafel slope @ 10mA cm^−2^, in acidic solution [86]. Interestingly, the cage-confinement pyrolysis protocol has been adopted to fabricate ultra-small WC nanoclusters/NPs as reported by Xu Yan-Tong and his co-workers. An RHO-[Zn(eim)_2_] type zeolitic metal azolate with hydrophobic nanocavities (d = 1.84 nm) framework MAF-6, Heim = 2-ethylimidazolate, possessing large nanocages and small apertures (average diameter = 0.76 nm), is chosen to restrain the metal source W(CO)_6_. WC nanocluster exhibits the least overpotential and Tafel slope of 51 mV and 49 mV dec^−1^ @ 10 mA cm^−2^ respectively. In addition the large exchange current density of 2.4 mA cm^−2^ has recorded which is quite high among all the molybdenum/tungsten oriented catalysts in acidic solution [87]. A facile one-step protocol to prepare metastable WC NPs from phosphotungstic acid (H_3_PW_12_O_40_) was established by Takafumi et al. The NPs with an average diameter of 1 to 50 nm dispersed over the C-substrate were prepared through a simple operation series that involved the incorporation of carbon black with H_3_PW_12_O_40_ followed by heat treatment at 1000 °C. The produced WC NPs exhibited an outstanding catalytic activity for HER, an onset potential of 147 @ 1 mAcm^−2^, with the least Tafel slope of ~50 mV dec^−1^ [88]. Tang Chaoyun and his team investigated the WC-supported Pt electrocatalyst using the microwave-assisted ethylene glycol process. The tungsten carbide-supported Pt electrocatalyst featured a small onset overpotential of ~100 mV @ 1 mA cm^−2^, Tafel slope of 73 mVdec^−1^, and an exchange current density of 0.241 mA cm^−2^ [89].

#### 3.2.2. Tungsten Carbide and Its Composites

Zheng et al. prepared WC_x_/C composite by a combined solution combustion synthesis (SCS) method integrated with in-situ carbothermal reduction reaction. The WC_x_/C composite exhibited a good HER catalytic activity, with η_10_ of −264 mV and Tafel slope of 85 mV dec^−1^ @ 10 mA cm^−2^ in 0.5 M H_2_SO_4_ [90]. A 2D W_2_C single crystal incorporated graphene (2D WC-graphene) has been prepared through three steps by Mengqi et al. Initially, a Ga droplet is placed on W substrates at the center region of the quartz tube. Secondly, Ga-W substrates was heated to 980–1020 °C at a rate of 30 °C/min under the combined flow of Ar and H_2_; in the last step the substrates were exposed to carbon source (methane (CH_4_)) for 30 min under Ar/H_2_ resulting in the growth of 2D WC-graphene in-plane heterostructures. The overpotential of the catalyst is very small (120 mV @ 10 mA cm^−2^) and the determined Tafel slope is 38 mV dec^−1^ in an acidic solution [91]. Han Lei and coworkers studied the formation of nitrogen-doped carbon spheres encapsulated by W_2_C nanocrystallites using a combined in situ polymerization and carburization process. They recorded the overpotential of 290 mV in acidic solution and bit higher of 300 mV in alkaline solution @ 10 mA cm^−2^ for the resultant catalyst. In addition, the catalyst has exhibited the Tafel slopes of 110 mV dec^−1^ and 133 mV dec^−1^ in acidic and alkaline media, signifying the supportable HER kinetics in acidic medium [92]. W_2_C and tungsten disulfide (WS_2_) have been constructed on vertically aligned MWCNT forests in recent years. Initially an ultra-thin layer of tungsten been placed on vertically aligned MWCNT forests followed by heating at 770 °C under inert atmosphere or under sulfur-vapor for getting W_2_C or W_2_S, respectively. The WC-CNT and WS_2_-CNT composites have the overpotentials as low as 489 mV @ 10 mA cm^−2^, 689 @ 10mA cm^−2^ and the Tafel slope value 122, 182 mV dec^−1^ in 0.5 MH_2_SO_4_ [93]. Qiufang et al. reported a facile two-step methodology to fabricate ultra-small and phase-pure W_2_C particles supported on MWCNTs (W_2_C/MWCNT). W_2_C/MWCNT exhibited impressive electrocatalytic HER performance, featuring a small onset overpotential of ~50 mV @ 1mA cm^−2^, 123 mV @ 10mA cm^−2^, Tafel slope of 45 mV dec^−1^, and long-term cycling stability [83].

**Figure 6:**
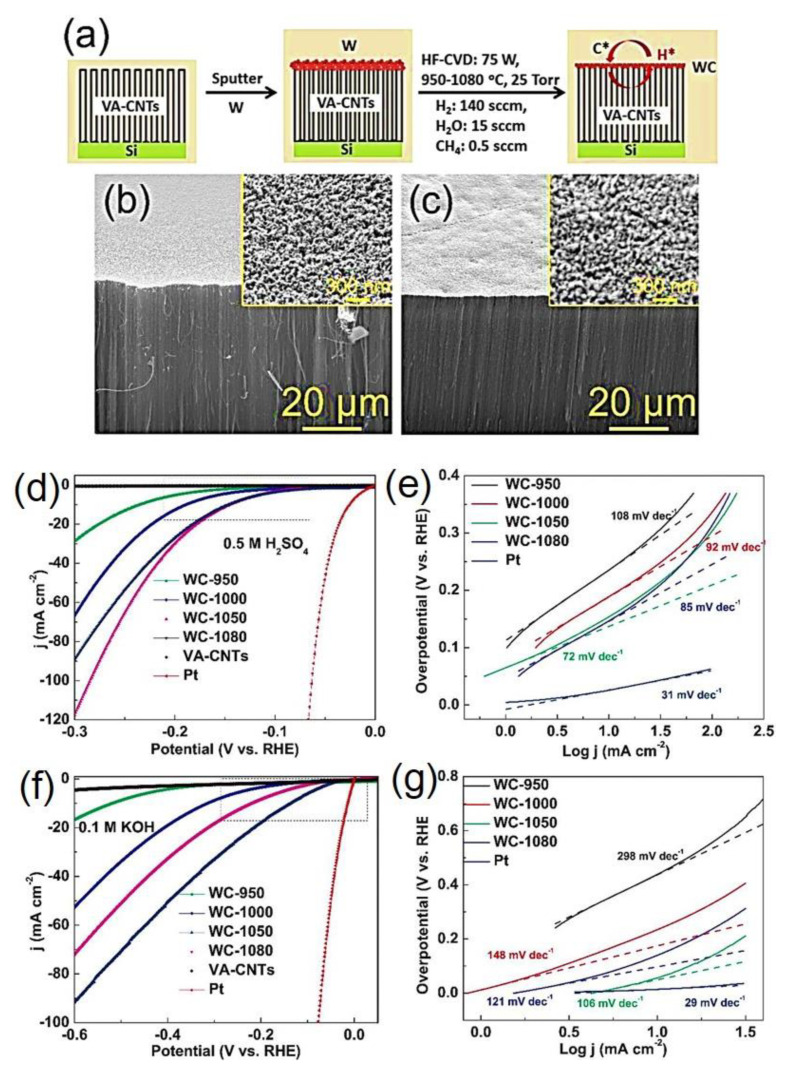
(**a**) Schematic of the formation of WC nanocrystals over VA-CNTs, (**b**,**c**) Field-emission SEM image of bare VA-CNT and WC nanocrystals over VA-CNT that formed at 1050 °C for 6 h, (**d**,**e**) LSV and Tafel slope plots of WC nanocrystals over VA-CNT at different temperatures in an acidic medium, (**f**,**g**) LSV and Tafel slope plots of WC nanocrystals over VA-CNT at different temperatures in an alkaline medium [94]. Copyright 2015 The American Chemical Society.

Recently, Xiujun et al. developed WC nanocrystals via sputtering as well as HF-CVD. These NCs were grown over vertically aligned CNTs by a two-step process as displayed in Figure 6 (W_2_C-CNTs). The corresponding SEM image confirming the formation of WC nanocrystals over the VA-CNTs (Figure 6b,c) and it exhibit an excellent catalytic activity for HER in both alkaline and acidic solutions as shown in Figure 6d–f [94]. The overpotentials (η_10_) in acid solution drives a current of 10 mA cm^−2^ of 145 mV, onset potential of 15 mV, exchange current density @ 300 mV of 117.6 mV and Tafel slope values of 72 mV dec^−1^. Further, for alkaline media (η_10_) of 137 mV, onset potential of 16 mV, exchange current density @ 300 mV of 33.1 mV, and Tafel slope of 106 mV dec^−1^ were observed [94]. The electrochemical characteristics of various W_2_C/WC-based electrocatalysts for improved HER activity are listed in Table 2.

Due to their ideal chemical reactivity and tunability, 2D-TMCs are largely used as the potential supports for nanostructured particle catalysts and that the existence of nobel metal nanoparticles favors the reduction of M constituent of the MXene (M_n+1_X_n_T_x_) and eliminate the surface functional groups effectively [95]. The further reduction of these supports could be led to form metal components in the supports to nanoparticle which in turns to contact with the surface of the supports thereby leading to form a well-ordered intermetallic compound material via reactive metals support interactions (RMSIs) in few cases. RMSI denotes to the chemical reaction of the support and metal that stimulates the bimetallic nanostructures’ formation, which is determined through the superior thermodynamic stability of the subsequent intermetallic compounds. MXenes could easily favors this process by means of its 2D structures with M-C bonds. This M-C bonds are usually weaker than that of M-O bonds in a characteristic oxide supports. Owing to this enhanced chemical reactivity, it permits RMSIs to ensue at very low temperature and, so, facilitate the control of size of the particle in contrary to high temperature reduction that necessary for early bulk carbides and transition metal oxides [96,97]. In few cases, over MXenes intermetallic compounds might be produced that is impossible in conventional carbide or oxide supports and their complete physicochemical properties could be controlled through in-situ chemical reduction at adequate temperature. Forming these types of supports with ideal reactivity and chemical stability is still a challenging task. Recently Li et al. have investigated the formation of Pt_3_Ti and Pt_3_Nb intermetallic compounds through the RMSIs on Pt/Ti_3_C_2_T_X_ and Pt/Nb_2_CT_x_ catalysts and reported elsewhere [81].

## 4. Summary and Future Outlook

TMCs especially Mo_2_C and WC/W_2_C are considered as the perfect alternatives to noble metal catalysts, such as Pt, Pd, and Au, for HER, although their HER catalytic activity is restricted. Different synthesis protocols and catalyst design strategies limit the variation in catalytic activities between the noble metal and the Mo_2_C- and WC/W_2_C-based catalysts. Although Mo_x_C and WC/W_2_C-based compounds are well known catalysts in chemical engineering, the research on their function as electrocatalysts is still in its primary stage. This review is focus on the recent development of Mo_x_C and WC/W_2_C-based electrocatalysts for HER activity. One of the major concerns in designing and fabricating the Mo_x_C and WC/W_2_C-based electrocatalysts is the low-density of active sites. Moreover, structural collapse and particle agglomeration occur at high carburization temperatures, leading to the formation of bulk materials. To overcome these drawbacks, diverse nanostructure engineering methods have been reported. Doping and nanostructuring have emerged as vital tools for altering the properties and behaviors of these catalysts. However, issues, such as mass production of catalysts, understanding the mechanism of HER activity, and controlling the nanostructure, still need to be addressed.

Primarily, the excellent HER catalytic activity of the recent metal carbide catalysts alone is not suitable for their practicability. Various synthetic protocols have been developed to control the composition and nanostructure of these electrocatalysts, and further enhancements in the catalytic activity of these catalysts are required. Doping heterometal atoms or carbon supports, such as CNT, RGO, and graphene, could efficiently enhance the performance of the Mo_2_C and WC/W_2_C-based catalysts. Recently, MOF-derived catalyst designing strategies have been in the limelight and could be prolonged to fabricate Mo_2_C and WC/W_2_C-based electrocatalysts with refined stereo-structures and components. Emerging innovative or new-fangled templates can also support the construction of hierarchical nanostructures, including hollow structured or core-shell WC/W_2_C and Mo_2_C-based composites. In addition, these metal carbides can be integrated with other potential efficient compounds such as metal sulfides, phosphides, and selenides to make multicomponent catalyst systems to improve the overall catalytic activity.

Second, extensive and widespread efforts are required to merge the experimental and theoretical evaluations in order to understand the correlation between the physicochemical structures and the electrochemical behaviors of the Mo_2_C and WC/W_2_C-electrocatalysts. Advancements in catalyst structures and examination of the active site origins can aid in the atomic-scale study of the growth and nucleation processes of metal carbides catalysts. More systematic and detailed theoretical studies on the electrochemical HER activities of the Mo_2_C and WC/W_2_C-based electrocatalysts are necessary. These attempts and efforts would guide the scientific community to better tune the chemical and physical properties of the Mo_2_C and WC/W_2_C-electrocatalysts.

In a nutshell, the mass production of hydrogen from water electrolysis is known to be a highly challenging task, and we still need to go a long way to achieve this goal. It majorly depends on the accessibility of cost effective electrocatalysts. However, the reported well-designed, morphology controlling synthesis methods may not be appropriate for the large-scale production of such catalysts. Hence, the growth of cost-effective and scalable processes for large-scale fabrication of Mo_2_C and WC/W_2_C-based electrocatalysts are essential.

## Figures and Tables

**Table 1:** Electrochemical characteristics of various Mo_2_C-based electrocatalysts for improved HER activity.

Catalyst	Synthesis	Morphology	*η*_j_(mV)	*j*(mA cm^−2^)	Tafel Slope (mV dec^−1^)	Ref
N-Mo_2_C NSs	CVD	nanosheets	99	10	48.3	[44]
Mo_2_C-RGO	Hydrothermal carbonization	nanoparticles	70	10	57.3	[45]
β-Mo_2_C	Hydrothermal/calcination	nanospheres	240	10	120	[46]
Mo_2_C/CC	Hydrothermal/calcination	Nano-island	140	10	124	[47]
Mo_2_C	Chemical activation process	nanoparticle	240	20	56	[48]
β-Mo_2_C/C	hydrothermal	Irregular ill-defined particles	330	20	72	[49]
Mo_2_C/CNT	In situ-carburization	nanoparticle	64	1	63	[50]
β-Mo_2_C	Hydrothermal/calcination	nanoparticle	165	10	55	[51]
MoC-Mo_2_C	Controlled carbonization	nanoparticle	33	10	42	[52]
Mo_x_C	One-pot pyrolysis	nanobelts	50	10	49.6	[53]
3DHP-Mo_2_C	Scalable salt-template process	Highly interconnected 3D porous network	75	1	75	[55]
Mo_2_C/NCF	High temperature calcination	nanoflowers	144	10	65	[57]
Mo_2_C/C	pyrolysis	nanosheets	180	10	72	[59]
Mo_2_C-G	In situ-carburization	nanoparticle	150	10	57	[61]
Mo_2_C/CLCN	Hydrothermal/calcination	nanorods	155	10	48.2	[66]
Mo_2_C/NC	Polymerization/carbonization	sprout	140	10	114.4	[67]
1D Mo_2_C	In situ-carburization	nanosheets	36	10	47	[69]

RGO—reduced graphene oxide; G—graphene; CNT—carbon nanotube; NSs—nanosheets; NC—nitrogen doped carbon; 1D—one dimensional; 3DHP—three dimensional highly porous network; NCF—nitrogen doped carbon framework; *η*_j_—overpotential; *j*—current density.

**Table 2:** Electrochemical characteristics of various W_2_C/WC-based electrocatalysts for improved HER activity.

Catalyst	Synthesis	Morphology	*η_j_*(mV)	*j*(mA cm^−2^)	Tafel Slope (mV dec^−1^)	Ref
W_2_C/MWCNT	carburization	nanoparticles	123	10	485	[83]
W_2_C-thinfilm	CVD	nanograins	263	10	42.2	[84]
W_2_C	Plasma assisted carburization	nanowires	118	10	55	[85]
WC	Plasma assisted deposition	nanowall	160	10	67	[86]
WC	Cage confinement pyrolysis	nanoparticle	51	10	49	[87]
W_2_C	High temperature calcination	nanoparticles	368	20	50	[88]
Pt/WC	High temperature calcination	Spherical particles	22	10	28.8	[89]
WC_x_/C	Combustion reaction	nanoparticle	264	10	85	[90]
2D WC-G	Liquid metal solvent-based co-segregation strategy	Single crystals	120	10	38	[91]
N-doped WC	In-situ polymerization/carburization	nanospheres	290	10	110	[92]
WC/CNT	CVD	nanoflakes	435	10	103	[93]
W_2_C	carburization	nanoparticles	50	10	45	[83]
W_2_C	HF-CVD	nanocrystal	117.6	10	72	[94]

G—graphene; CNT—carbon nanotube; NSs—nanosheets; NC—nitrogen doped carbon; CVD—chemical vapor deposition; HF-CVD—high filament chemical vapor deposition.

## Data Availability

Not applicable.
